# Atomic-Layer-Deposition-Assisted Interfacial Engineering of Metal-Oxide-Metal Devices via Multilayer Structures to Improve Leakage Characteristics

**DOI:** 10.1021/acsomega.4c07424

**Published:** 2025-04-24

**Authors:** Zhao-Cheng Chen, Hao-Jung Liu, Yu-Chi Chang, Shoou-Jinn Chang

**Affiliations:** † Institute of Microelectronics, Department of Electrical Engineering, National Cheng Kung University, Tainan 70101, Taiwan; ‡ Department of Engineering Science, National Cheng Kung University, Tainan 70101, Taiwan

## Abstract

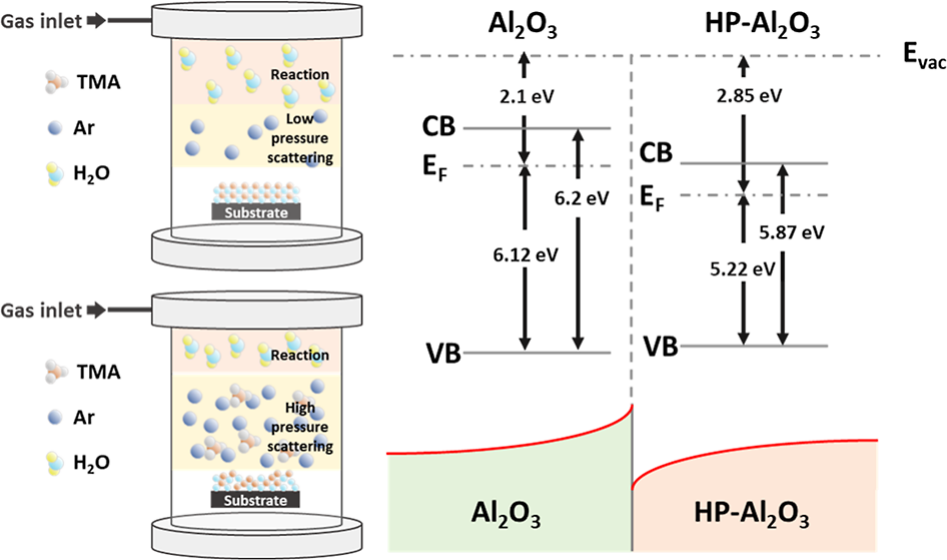

The defect-controlled charge transfer mechanism in insulators is crucial for advanced electronic devices. In this study, metal/insulator/metal devices with a multilayer stacked structure are developed in which the off-state current value is reduced by 1 order of magnitude compared to a single-layer structure by modulating the deposition conditions of the atomic layer deposition system. The results of x-ray photoelectron spectroscopy suggest that the pressure modulation of the atomic layer deposition system drives the formation of hydroxyl groups. The different band structures formed by such an oxide layer with more hydroxyl groups further affected the current transport. The possible pathways for carrier transport are presented in detail through electrical analysis and provide the potential for different energy band multilayer stacked structures as advanced electronic devices.

## 1 Introduction

Following the continuous pursuit of improved performance and functionality of advanced electronic devices, the role of defects in insulators in charge transfer mechanisms has increasingly aroused concern. Insulators, as pivotal components in electronic devices, have defects that are critical to current transfer and device performance. The research revealed that the reliability and performance of electronic devices can be significantly improved by relatively controlling defects in the insulation layer.^1−4^ However, despite the advances in coating technology, the application of defect engineering of an insulator to advanced electronic devices is still subject to unavoidable challenges, mainly involving issues such as the accuracy and reproducibility of defect control, the high dependence on process parameters, and the complex mechanisms by which defects affect the performance of the devices.

Atomic layer deposition (ALD), which has a unique approach of controlling the deposited thin film with angstrom-level precision, has been recognized as an essential technology for film coatings in the semiconductor process. Because of the growth mechanism of self-limiting surface reaction,^5^ ALD enables the formation of high film quality with high step coverage and compactness^6^ and achieves an excellent conformal nature in high aspect ratio structures. Recent studies have indicated that ALD process parameters (including the precursor pulse time, purge time, and deposition temperature) play a critical role in the formation and characterization of the prepared films. In particular, the adjustment of process pressure affects the surface reaction saturation^7^ and defects/impurities,^8^ which in consequence changes the physical and chemical properties of the films. Lee et al. have caused the conduction band offset of the material to control the defect positions of the insulating layer in the MIOS diode through the sputtering system, thus enhancing the rectification performance of the diode.^9^

In this study, the tuning of the energy level in the insulating layer via the ALD technique is dedicated to where it is crucial to investigate its effects on the charge transfer mechanism. By tuning the deposition conditions of the ALD system, which performs defect engineering at specific locations for different process pressures, the dielectric layers with a total thickness fixed at 20 nm are fabricated and multilayer stacked metal/insulator/metal (MIM) structures are developed. The *I*–*V* characteristics were evaluated and confirmed that the multilayered interfaces could effectively suppress the off-currents, which helps to increase the charge carrier trapping and improve the reliability and stability of the device. The mechanism for carrier injection has revealed the potential for controlling the defect engineering in the insulator layer. The proposed process approach for ALD-based modulation of defect engineering can provide a new design strategy for optimizing the reliability of electronic devices.

## 2 Experimental Details

First, an indium tin oxide (ITO)/glass substrate was cleaned with acetone, methanol, and deionized water in an ultrasonic bath for 15 min to remove organic residues. After that, the liquid droplets on the surface of the substrate were blown away by nitrogen gas and dried in an oven at 40 °C. The size of all samples was 20 × 15 mm^2^. Subsequently, Al_2_O_3_ films were deposited on the surface of the ITO/glass substrate through an ALD process using trimethylaluminum (TMA, 99.999%) and deionized water as the aluminum metal and oxygen precursors, and Ar as a carrier gas was employed. The Ar flow was set at 500 sccm to remove unreacted precursors and any byproducts. The temperature of the two precursors was retained at room temperature in precursor cylinders and transported to the reactor under 25 sccm of Ar gas (99.99%). All delivery lines were maintained at 80 °C to prevent precursor condensation. The Al_2_O_3_ films were subjected to 150 °C with a deposition cycle of TMA pulse of 0.1 s → Ar purge of 10 s → H_2_O pulse of 0.1 s → Ar purge of 10 s, where the thickness of the Al_2_O_3_ was up to 20 nm. To investigate the impact on the electrical performance of the process pressure, the Al_2_O_3_ films were deposited with the ALD valve opening adjusted, and other deposition parameters were maintained unchanged. The chamber pressure in the reactor during the deposition process was 0.1 Torr in the low-pressure process, while in the high-pressure process, the pressure in the reactor was adjusted to 1 Torr by tuning the valve opening. After deposition, the Al_2_O_3_ thin films were postannealed at an annealing temperature of 250 °C under atmospheric conditions. Aluminum was deposited on the Al_2_O_3_ film by sputtering using the shadow mask to define the patterns of top electrodes. The resulting electrical characteristics were measured using a Keithley 2636 semiconductor sourcemeter. Elemental analysis of Al_2_O_3_ thin films was carried out by using X-ray photoelectron spectroscopy (XPS, ESCA PHI 5000 VersaProbe).

## 3 Results and Discussion

**Figure 1:**
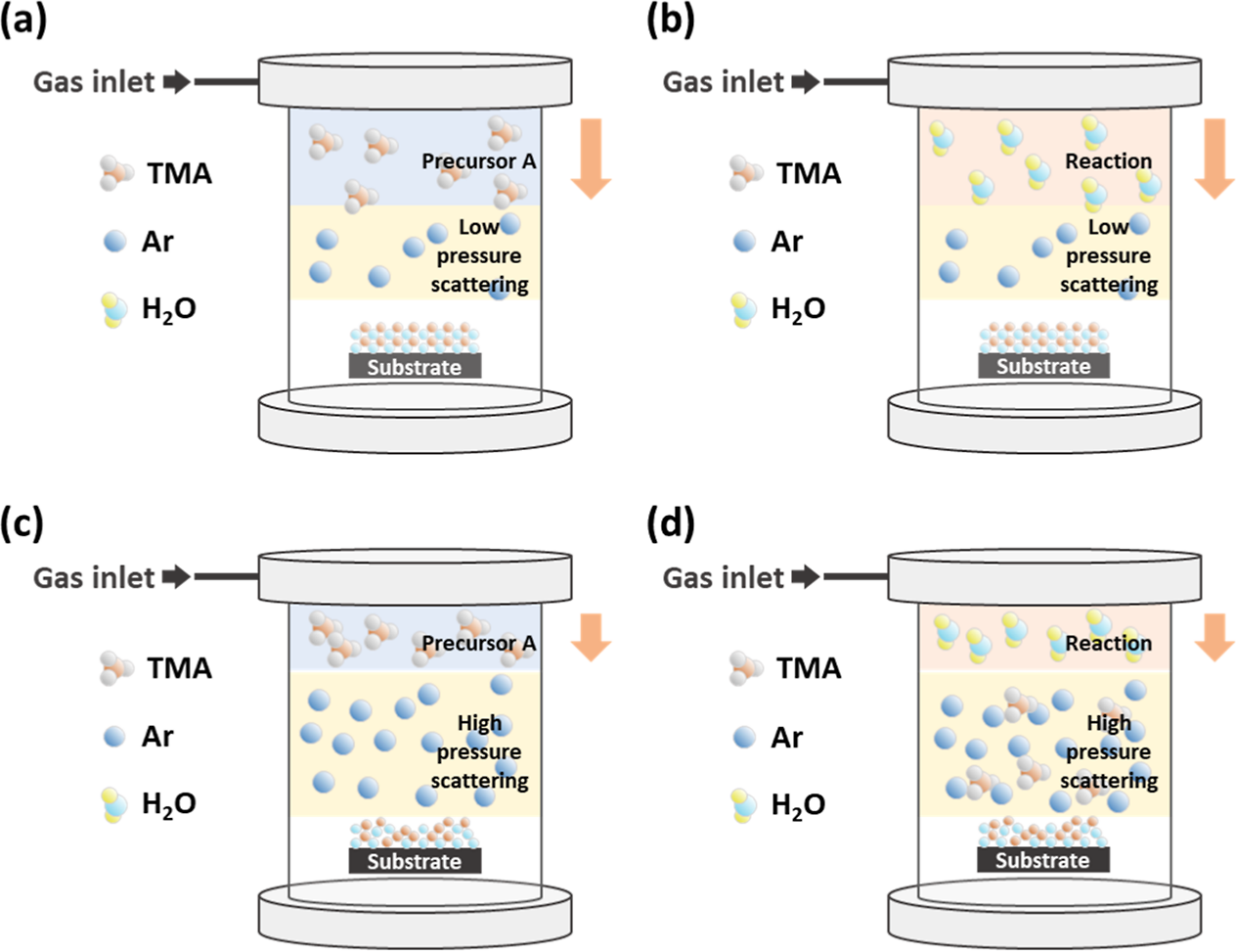
Schematic diagram of the ALD process with the (a) precursor and (b) reactants at low operating pressure and the (c) precursor and (b) reactants at high operating pressure.

In cutting-edge nanofabricated thin film coating technology, the ALD process parameters are correlated to the quality and functionality of the thin film. To realize precisely controlled defects, the efficiency of the reaction chamber with pumping is elaborately varied by the adjustment of the pump extraction valve. The illustration in Figure 1depicts the sequence of pulse and purge in the ALD process at low and high operating pressures. At low operating pressures, the precursors and reactants are introduced to flow sequentially over the substrate surface, and deposition is accomplished by surface reactions. Initially, the TMA precursor is introduced into the reactor for limited reactions with the –OH groups on the substrate surface to reach saturation,^10^ followed by a purge step in which the TMA residues and excess unreacted byproducts are evacuated from the reactor. Subsequently, the reactants are introduced to react with the functional groups on the substrate surface and further removed from the reactor. In contrast, process conditions with high operating pressures are subject to the effects of residual gases in the reactor, which prevent the precursors and reactants from approaching the substrate surface, resulting in a difference in the cleanliness of the reactor chamber during the purge step. The reactants entering the chamber are exposed to unreacted TMA and reaction byproducts which are susceptible to reacting before reaching the substrate and the reaction between the postreaction complex and the surface species on the substrate that may influence the deposition type of the film.^11,12^ The film growth reaction is expected to transition from chemisorption to chemisorption with some physiosorbed reaction mechanism, resulting in significant differences in the deposition rate and properties of the film. This is indicated by the subsequent results of the refractive index and the growth per cycle (GPC) of the film.

**Figure 2:**
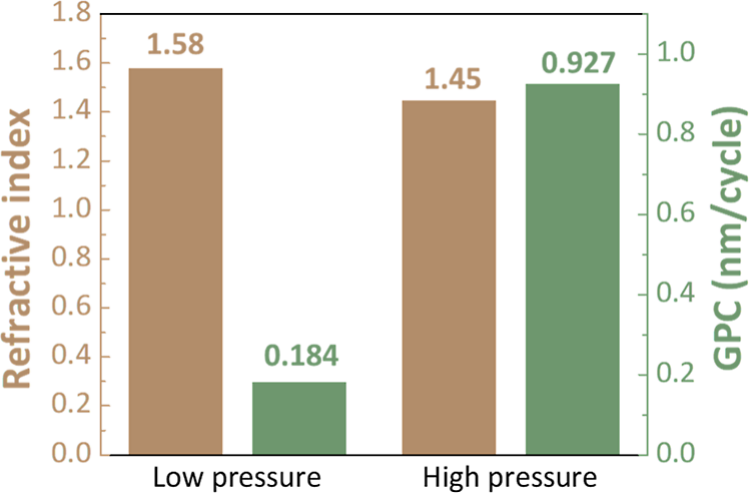
Refractive index and GPC of Al_2_O_3_ films were prepared by ALD at different operating pressures.

Figure 2 depicts the refractive index and GPC of the deposited films at 632.8 nm wavelength by an ellipsometer at high process pressure as well as at low process pressure. The refractive index and density of ALD-Al_2_O_3_ films were established by the Lorentz–Lorenz relationship.^13−15^ As the process pressure increases, the refractive index declines from 1.58 to 1.45; therefore, it can be inferred that the density of Al_2_O_3_ films decreases slightly, while the process pressure increases. In addition, the GPC varies from 1.84 Å/cycle to 9.27 Å/cycle as the process pressure increases, indicating that the growth rate of the films differs as a result of the pressure variation. Previous literature has indicated that the process pressure for ALD is closely related to the flow rate in the chamber and that increasing the process pressure necessitates higher gas flow rate to realize more diffusive species transport.^16,17^ On the other hand, prolonged purge time would prevent the precursors to outgas or react prematurely during the reactant exposure step.^18^ The internal composition of films is significantly influenced by process conditions with fixed chamber flow rates and purge times.

**Figure 3:**
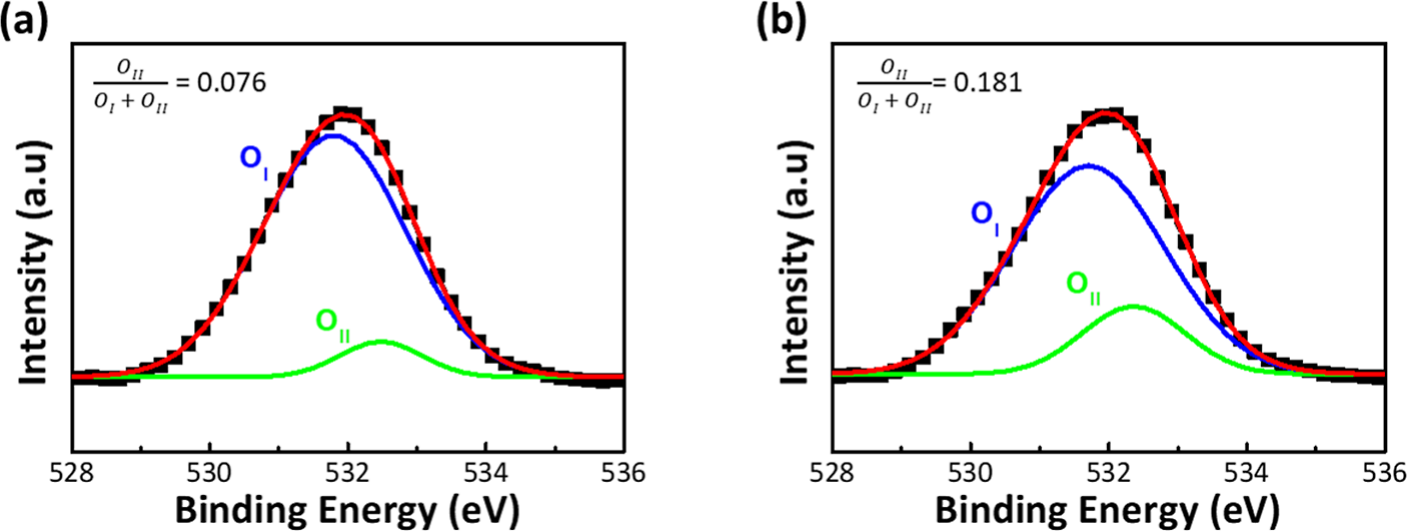
XPS O 1s peaks of Al_2_O_3_ films fabricated with (a) low pressure and (b) high pressure.

To explore the changes in the chemical composition of Al_2_O_3_ films which was obtained at two process pressures, XPS analysis was performed to identify the results as illustrated in Figure 3a,b. The O 1s core-level spectra can be deconvolved into a Gaussian–Lorentzian function with two components: the peak located at 531.7 eV (O_I_) from metal–oxygen bonding (Al–O) and the peak with a binding energy of 532.5 eV (O_II_) originating from hydroxyl groups. The relative peak intensity [O_II_/O_I_ + O_II_] increases from 0.076 to 0.181 when the operating pressure rises from 0.1 to 1 Torr, which is attributed to the large amount of precursors filling the entire reaction space. The reactants were subsequently dosed into the reactor, which contained both the residual precursors and the newly injected reactants. In which case, the residual precursors react with the reactants resulting in chemisorption reaction followed by further reactive reaction with the substrate surface, so that the reaction condition on the substrate surface was varied from the original ALD process based on self-limiting reaction coupled to a CVD-like growth reaction.^19,20^ While the deposition rates increased, more defects are produced in the film.^21−23^

**Figure 4:**
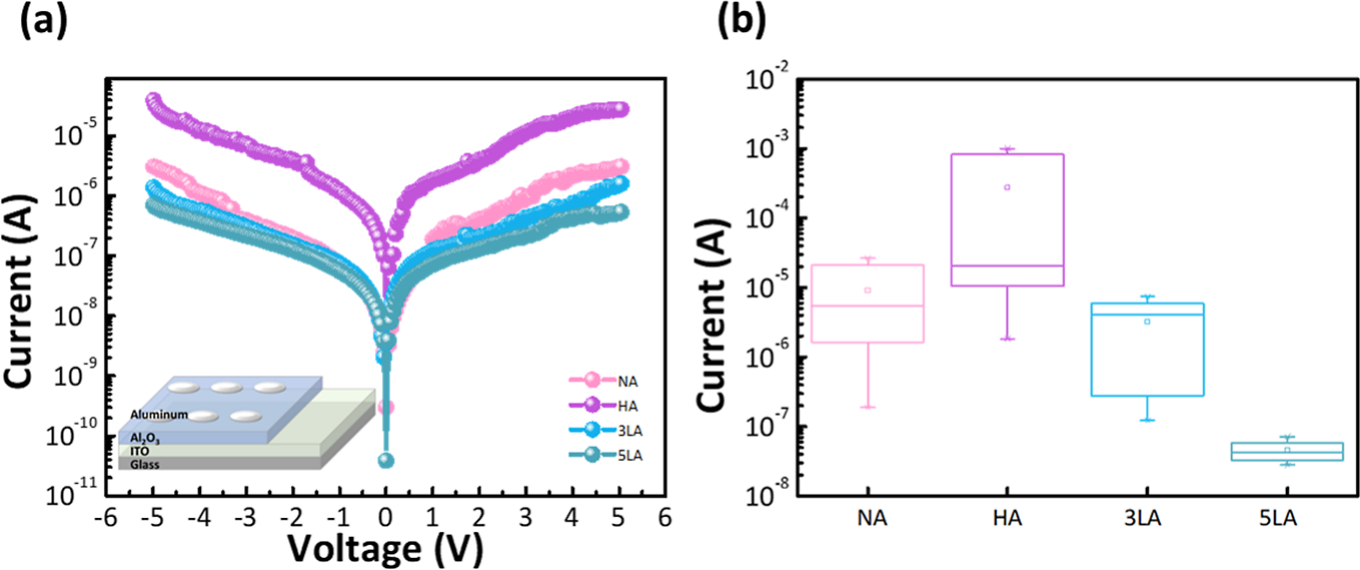
(a) Current–voltage characteristics of MIM devices with different numbers of insulating layer stacks (the inset in (a) shows the schematic structure of the MIM device consisting of ITO/insulator 20 nm Al_2_O_3_/aluminum). (b) Current organization of the device at 1 V.

To adequately investigate the influence of the process pressure of ALD on the device, four structures of insulators were designed with a constant total thickness of 20 nm by tuning the process pressure to fabricate the Al_2_O_3_ thin films. Therefore, the Al_2_O_3_ layers prepared at (1) low pressure and (2) high pressure are labeled as NA and HA samples, respectively. The stacked films fabricated at different pressures were constructed as (3) low-pressure Al_2_O_3_/high-pressure Al_2_O_3_/low-pressure Al_2_O_3_ and (4) low-pressure Al_2_O_3_/high-pressure Al_2_O_3_/low-pressure Al_2_O_3_/high-pressure Al_2_O_3_/low-pressure Al_2_O_3_, which were designated as 3LA and 5LA, respectively. The inset of Figure 4a presents MIM devices composed of an Al/Al_2_O_3_/ITO structure. It was revealed by *I*–*V* measurement, depicted in Figure 4a, that the off-current value of the NA device (1.9 × 10^–7^ A) was lower than that of the HA device (1.82 × 10^–6^ A) at a bias voltage of 1 V, with a difference of approximately 1 order of magnitude, when the total thickness of the insulating layer remained constant. Afterward, the electrical performance of the 3LA device and the 5LA device was confirmed, in which the multilayer structure exhibited significantly reduced current values (3LA: 1.23 × 10^–7^ A vs 5LA: 7.19 × 10^–8^ A). The leakage current values measured by applying bias voltages decrease with the increase in the stacked layers. Mainly, the current value of the 5LA device is decreased by close to 2 orders of magnitude compared to the single-layer NA device, probably resulting from the layer-to-layer interface that blocks the current penetration through the insulating layer.^24,25^Figure 4b organizes the current values of the four devices measured several times at a 1 V bias. In the single-layer structure, the HA device exhibits high leakage characteristics and less stable current performance than the NA device. However, in the stacked structure, the leakage performance tends to decrease as the number of stacks increases. Among them, 5LA has the best leakage performance and current stability. This indicates that the multilayer stacked structure can reduce the leakage characteristics of dielectric films. Thin films deposited alternately under two deposition conditions, low pressure and high pressure, are the main factors affecting the leakage current. The appropriate introduction of multilayer structure can efficiently reduce the leakage current, which follows a similar trend with other multilayer structures.^26^

**Figure 5:**
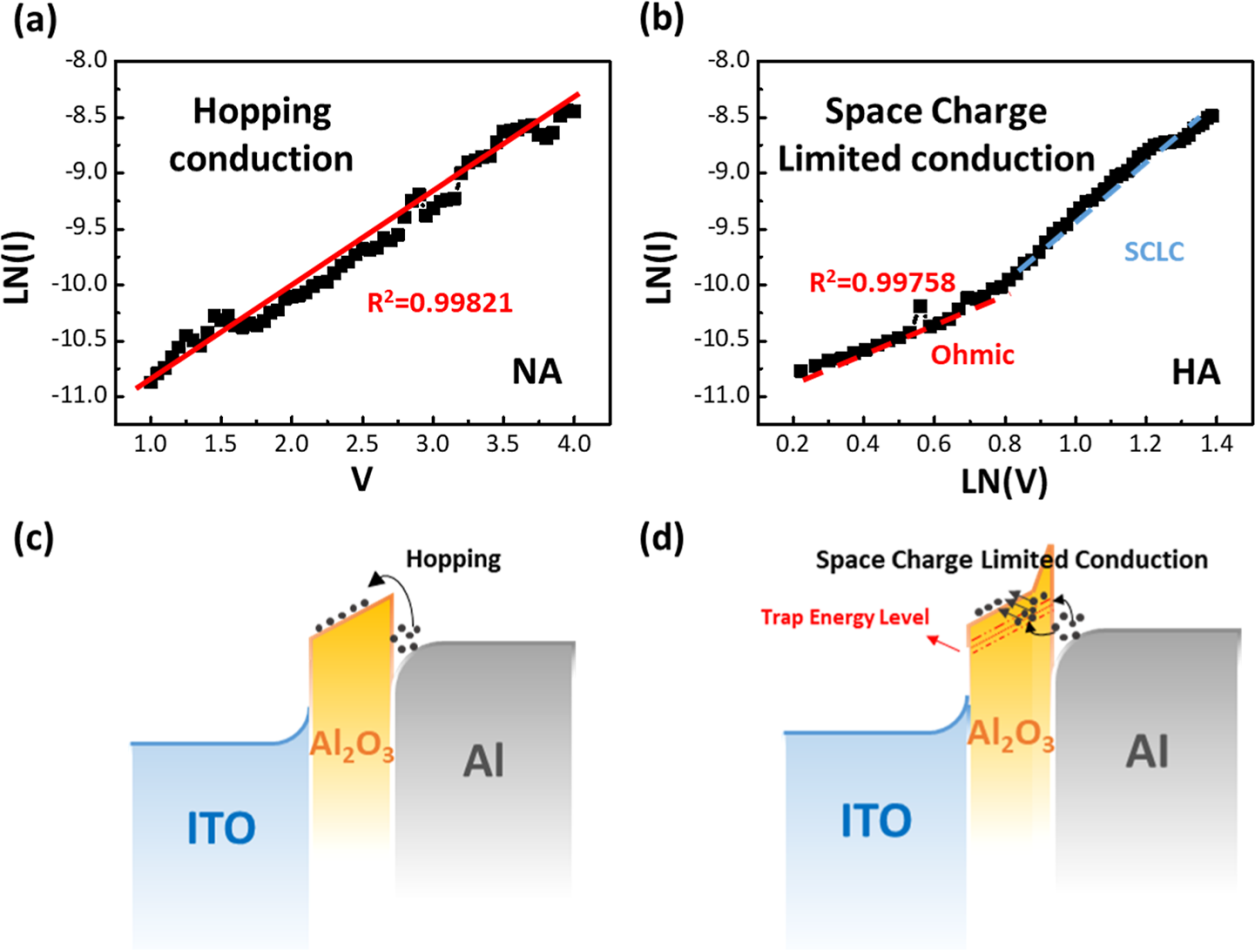
Current transport characteristics of the MIM structure and its corresponding mechanism. The fitting *I*–*V* characteristic curves of the devices prepared during a (a) low-pressure process (NA device) and (b) high-pressure process (HA device). Modeling of defect-induced current flow of the (c) NA device and (d) HA device.

In view of verifying the current transport mechanism in the insulating layer, the charge conductive mechanism of the fabricated structures under different process pressures was modeled by fitting the *I*–*V* curves, respectively. Figure 5a,c shows the conductive paths formed by highly energetic electrons migrating to defect states in the Al_2_O_3_ insulating layer of the device at low operating pressures, which is in accordance with the hopping conduction mechanism. In addition, Figure 5b,d displays the results of *I*–*V* fitting results for devices with high operating pressures, where the conduction mechanism transforms from the typical ohmic conduction (*I* ∝ *V*_*m*_, *m* = 1) in the low electric field state to the space-charge-limited conduction (*I* ∝ *V*_*m*_, *m* > 1) mechanism as the electric field increases.^27^

**Figure 6:**
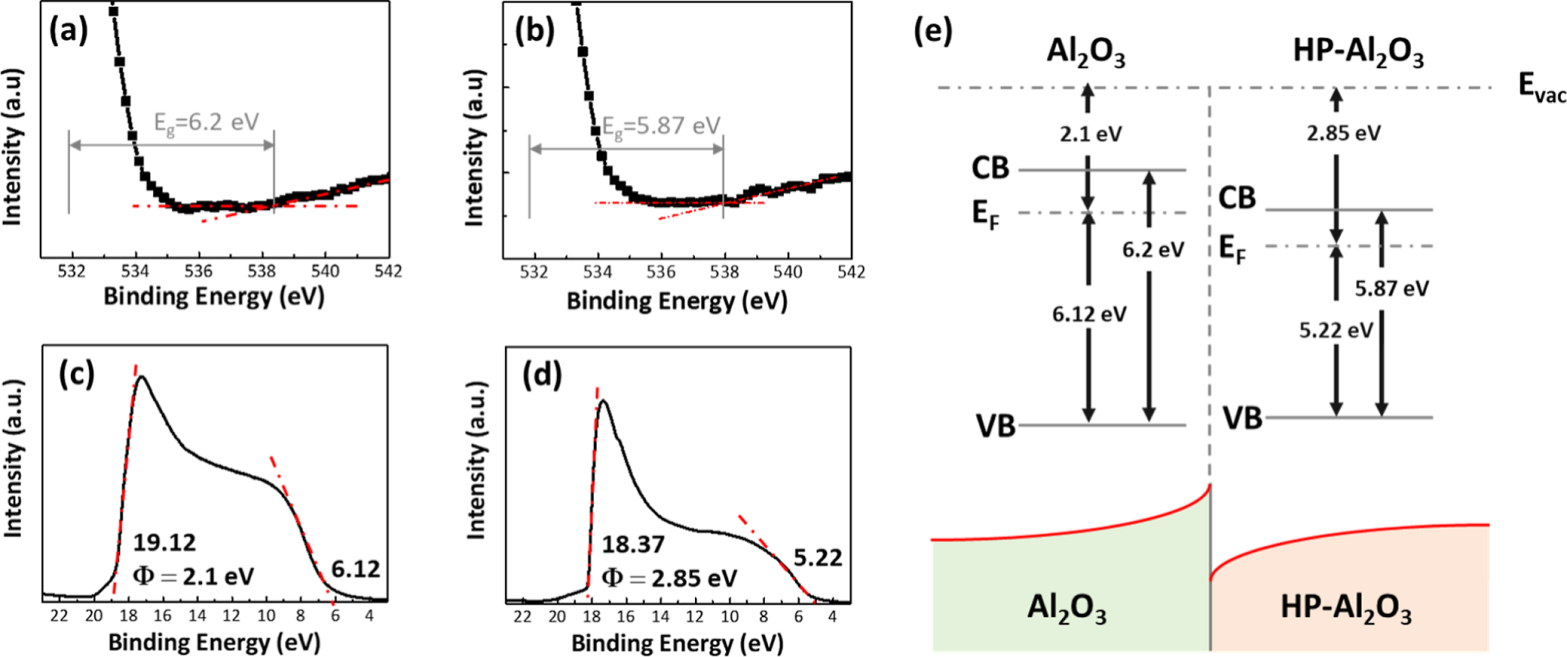
Optical analysis and energy band diagram of Al_2_O_3_. The band gap of the (a) NA and (b) HA films from the O 1s peak obtained by high-resolution XPS. UPS spectra of (c) NA and (d) HA films. (e) Energy band diagram of NA and HA oxide films.

Figure 6a,b demonstrates the energy band gap (*E*_g_) of NA and HA films calculated from the inelastic loss phenomenon of the O 1s peak of the XPS spectrum,^28^ which are 6.2 and 5.87 eV, respectively. The photoemission spectra from the surface of the Al_2_O_3_ thin films were measured with ultraviolet photoelectron spectroscopy (UPS, light source He I, 21.22 eV)^29^ and by calculating the difference between the cutoff energy and the incident photon energy (21.22 eV), which was used as a basis for evaluating the work function of materials. Figure 6c,d reveals that the work functions of NA and HA films are 2.1 and 2.85 eV, respectively, and the energy differences between the Fermi level and the valence band edge are 6.12 and 5.22 eV, respectively. The energy band structures of NA and HA films were constructed based on the above analysis, as shown in Figure 6e.

**Figure 7:**
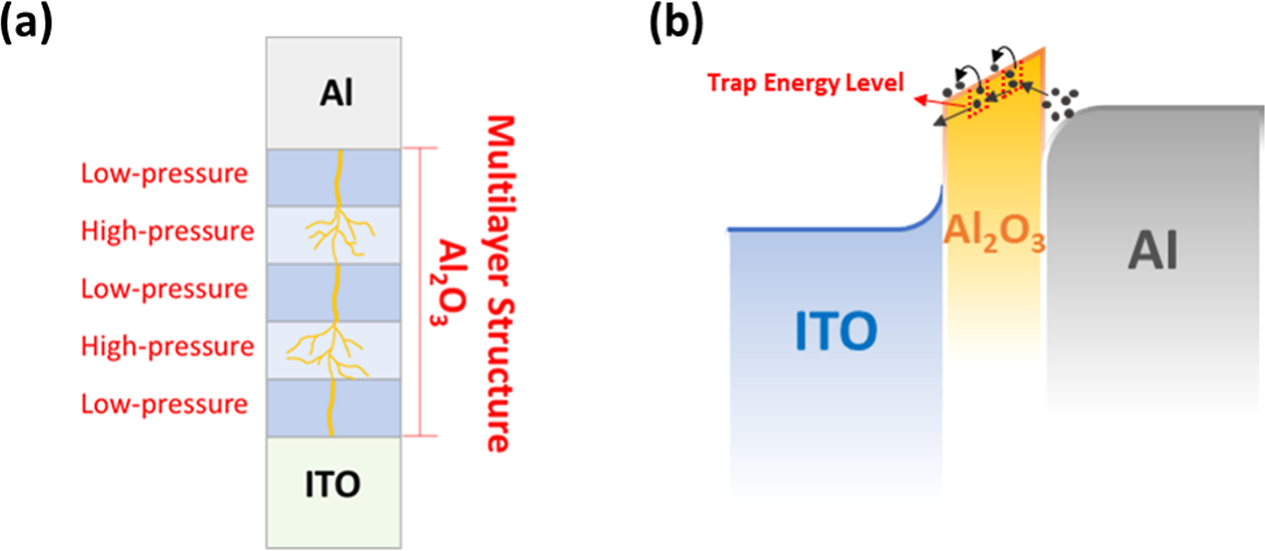
(a) Schematic diagram of a multilayer stacked structure that enables current transfer scattering and reduces off-current, and (b) modeling of defect-induced currents in a multilayer structure (5LA device).

In multilayer structures, where Al_2_O_3_ layers with different energy band structures were inserted to modulate the pressure increment, additional localized energy levels may be introduced in the energy gap, as shown in Figure 7. The energy level of Al_2_O_3_ which lies in the forbidden energy ranges acts as trapping sites for the available charge carriers, thus blocking the charge transfer.^30^ The presence of electron traps in the insulator may trigger the phenomenon of charge trapping when electrons flow through the insulator.^31^ The introduction of a multilayer structure with additional traps can prevent the effect of electron transfer because of the charge trapping phenomenon.^32^ Moreover, by introducing more traps into the multilayer structure, due to the presence of dangling bonds and incomplete atomic coordination, the conduction band at the interface between the film layers is discontinuous, which in consequence affects the position of the energy levels and thus reduces the leakage current of the multilayer structure.^33^

## 4 Conclusions

In conclusion, the charge transfer mechanism of insulator defect engineering stacked with multiple layers through the ALD technique has been verified. The process pressure is adjusted such that different energy band structures are produced at desired locations in the insulating layer, which in turn affects the current transfer. In addition, multilayer films with increasing numbers of stacked layers have considerably improved electrical performance compared to single-layer films, highlighting the suppression of leakage currents. The improvement in electrical properties is ascribed to the discontinuous band structure in multilayer films. A modeling of the hopping energy levels of charge carriers in Al_2_O_3_ thin films helps to get insight into the conductive mechanism. This strategy not only contributes to the improvement of the electrical performance of oxide electronic devices but also enables various functions to be realized by leveraging defects to address electronic device challenges.
